# Nocturnal water loss in mature subalpine *Eucalyptus delegatensis* tall open forests and adjacent *E. pauciflora* woodlands

**DOI:** 10.1002/ece3.44

**Published:** 2011-11

**Authors:** Thomas N Buckley, Tarryn L Turnbull, Sebastian Pfautsch, Mark A Adams

**Affiliations:** 1 Department of Biology, Sonoma State UniversityRohnert Park, CA; 2 Bushfire Cooperative Research CentreMelbourne, VIC, Australia; 3 Faculty of Agriculture, Food and Natural Resources, University of SydneySydney, NSW, Australia

**Keywords:** Alpine ash, Australia, *Eucalyptus*, fire, nocturnal transpiration, sap flux, snowgum

## Abstract

We measured sap flux (*S*) and environmental variables in four monospecific stands of alpine ash (*Eucalyptus delegatensis* R. Baker, AA) and snowgum (*E. pauciflora* Sieb. ex Spreng., SG) in Australia's Victorian Alps. Nocturnal *S* was 11.8 ± 0.8% of diel totals. We separated transpiration (*E*) and refilling components of *S* using a novel modeling approach based on refilling time constants. The nocturnal fraction of diel water loss (*f_n_*) averaged 8.6 ± 0.6% for AA and 9.8 ± 1.7% for SG; *f_n_* differed among sites but not species. Evaporative demand (*D*) was the strongest driver of nocturnal *E* (*E_n_*). The ratio *E_n_*/*D* (*G_n_*) was positively correlated to soil moisture in most cases, whereas correlations between wind speed and *G_n_* varied widely in sign and strength. Our results suggest (1) the large, mature trees at our subalpine sites have greater *f_n_* than the few Australian native tree species that have been studied at lower elevations, (2) AA and SG exhibit similar *f_n_* despite very different size and life history, and (3) *f_n_* may differ substantially among sites, so future work should be replicated across differing sites. Our novel approach to quantifying *f_n_* can be applied to *S* measurements obtained by any method.

## Introduction

It is often assumed that leaves stop transpiring at night, because stomata typically close in darkness in the laboratory (Meidner and Mansfield 1965; Sharkey and Ogawa 1987). Growing evidence suggests this assumption is incorrect (Benyon 1999; Donovan et al. 2001; Snyder et al. 2003; Bucci et al. 2004), and studies have reported nocturnal transpiration rates (*E_n_*) up to 25% of daytime rates for some tree species (Dawson et al. 2007). *E_n_* can have several important implications, including decoupling predawn leaf water potential from soil water potential (Donovan et al. 2001; Kavanagh et al. 2007) and altering integrated isofluxes of stable oxygen and carbon isotopes (Barbour et al. 2005; Seibt et al. 2007). It also poses a significant conundrum, because much of current theory of water use assumes water loss is beneficial for plants only insofar as it is coupled to carbon gain; if so, *E_n_* indicates a significant mechanistic limitation on plants’ ability to regulate water loss (Donovan et al. 1999; Daley and Phillips 2006).

It remains unclear how best to accommodate *E_n_* in estimates and predictions of forest water use. Indeed, most models for stomatal conductance are not structured to predict stomatal opening at night (e.g., Jarvis 1976; Ball et al. 1987), and *E_n_* is not well captured by current catchment models (Vertessy et al. 1997). This is partly because it is unclear whether the mechanisms of stomatal control are the same in the light and dark (Messinger et al. 2006; Barbour and Buckley 2007). Recent studies have begun to examine how hydraulic factors that influence stomatal opening in the light may also influence *E_n_*, including evaporative demand, soil moisture and wind speed, and other measures of atmospheric coupling (Rawson and Clarke 1988; Benyon 1999; Donovan et al. 2003; Bucci et al. 2005; Cavender-Bares et al. 2007; Dawson et al. 2007; Fisher et al. 2007; Moore et al. 2008; Phillips et al. 2010; Zeppel et al. 2010). The prevailing consensus is that *E_n_* is driven by evaporative demand and often regulated by soil moisture, whereas the effects of wind appear to vary among species and site conditions (Fisher et al. 2007; Phillips et al. 2010; Zeppel et al. 2010).

In light of the distinctive ecology of Australian vegetation, insights from other parts of the world may not always be applied to Australia with confidence. Yet, very few studies have examined *E_n_* in major Australian native forests over significant periods. Zeppel et al. (2010) recently quantified *E_n_* and its environmental correlates in *Eucalyptus parramatensis* and *Angophora bakeri*, in low-elevation, temperate open woodlands, and Phillips et al. (2010) studied eight eucalypt species in a common garden experiment. Both of these studies found relatively conservative *E_n_*, with the nocturnal fraction of diel water loss (*f_n_*) averaging 6–7% (cf. 0–20% among 27 woody species examined by Dawson et al. 2007). An earlier study on plantation-grown *E. grandis* (Benyon 1999) found similarly conservative *f_n_* (5%). Phillips et al. (2010) reported a strong effect of the product of wind speed (*U*) and *D*, whereas Zeppel et al. (2010) found an effect of *D* but not *U*. Additionally, Phillips et al. (2010) found greater *f_n_* when juvenile foliage was present. Any age effect may be compounded by height-related hydraulic suppression of stomatal conductance in taller trees (Ryan et al. 2006). Thus, one may expect more conservative nocturnal water use in taller trees; however, few relevant data exist for tall eucalypts.

We sought to test if these findings were also applicable in the high-elevation (1000–1700 m) forests and woodlands in southeastern Australia's Victorian Alps—the headwaters of catchments that supply water to millions of urban citizens and to much of the agricultural land in the region. While these catchments receive ca. 1200 mm of rainfall per annum, rain events are sporadic. These high-elevation catchments are vegetated with monospecific *E. pauciflora* Sieb. ex Spreng. (snowgum [SG]) woodlands and tall open forests of *E. delegatensis* R. Baker (alpine ash [AA]). SG and AA grow in close proximity to one another and in overlapping physiographic niches, yet display very different life histories and morphologies. At maturity, SG is much shorter (12–15 m) than AA (40–70 m). SG regenerates after fire mainly through epicormic or basal resprouts, whereas AA regenerates from seed. Despite the obvious economic and ecological importance of these species and the catchments that they dominate, nothing is known about nocturnal water loss in SG and AA, or how it is affected by atmospheric and soil drought.

We measured sap flux (*S*) with the heat-ratio method (HRM; Burgess et al. 2001) in relation to environmental variables in 23 mature individuals of AA (69 years old and 40–50 m tall) and SG (45–200+ years old and 12–20 m tall) growing in monospecific stands in protected national parkland near Falls Creek, Victoria, Australia, for an average of 1 year at each site. We compared sites using data from a 70-day period in which data were available from all sites. We developed a novel technique to separate the transpiration and refilling components of sap flux. Our objectives were to estimate the nocturnal fraction of diel water loss and its sensitivity to changes in soil moisture, evaporative demand, and wind speed in AA and SG, and to test the hypothesis that the much taller species (AA) would exhibit more conservative nocturnal water use. A secondary objective was to compare results for each species between two nearby sites of differing slope and aspect, as a pilot study to gauge the potential for site characteristics to cause variation in *f_n_*.

## Materials and methods

### Study sites

Our study sites are located near the town of Falls Creek (36°51′ S, 147°16′ E) in the Victorian Alps of Australia, approximately 230 km northeast of Melbourne. The two AA sites, AA1 and AA2, are located in mountainous terrain in the upper Kiewa Valley, roughly 5 km N/NW of Falls Creek, Victoria, in mature stands that germinated following a major stand replacement fire in 1939. AA1 is on a shallow, slightly south-facing slope (i.e., facing away from the sun), and AA2 is on a steeper, north-facing toe slope. The two SG sites, SG1 and SG2, are on the Bogong High Plains, roughly 10–15 km S/SE of Falls Creek, in mature stands. The age of these stands is unknown and individual trees may range in age from around 45 years of age (likely due to regeneration after fire) up to several hundred years. SG1 is on a moderate northeast-facing slope and SG2 is an isolated stand on a level site with greater exposure. Descriptive data for these sites are given in Table 1.

**Table 1:** Descriptive data for the sites used in this study. Environmental data are means for 12 November, 2009 – 20 January, 2010, the period for which data were available at all sites simultaneously

	*Eucalyptus delegatensis*		*E. pauciflora*	
		
Site name	AA1	AA2	SG1	SG2
Elevation/m	1300	1280	1600	1660
Slope/%	5–10	10–25	10–15	0
Aspect/°C	196	0	10	-
Approximate mean height/m	40	45	15	12
DBH range/cm	29.1–82.5	25.2–86.0	12.2–84.0	5.9–69.0
Mid 50% of DBH/cm	42.2–57.1	41.8–56.5	25.5–38	18.8–31.0
Air temperature/^o^C	15.0	15.9	13.3	12.5
Relative humidity/%	68.0	56.1	62.6	63.6
Soil RWC (5 cm)/%	28.1	17.1	29.3	19.3
Soil RWC (20 cm)/%	20.7	24.1	28.9	32.0
Soil RWC (50 cm)/%	19.3	21.9	21.2	27.8

RWC = relative water content.

### Atmospheric and soil measurements

Air temperature (*T_a_*/°C), relative humidity (RH/%), and soil relative water content (RWC/%) at 5-, 20-, and 50-cm depths were also measured within each site using sensors supplied by ICT International (Armidale, NSW, Australia). RWC was measured using standing wave soil moisture sensors (MP406, ICT Intl.). *T_a_*/RH sensors (HT1, ICT Int'l) were placed in radiation shields 1 m above the ground. Air water vapor mole fraction deficit (*D*, mmol mol^–1^) was calculated from *T_a_* and RH. Wind speed and above-canopy photosynthetically active radiation (PAR) were measured using HOBO weather stations (Onset Corp, Pocasset, MA). For SG1 and SG2, these stations were located in clearings less than 100 m from the measured stand of trees. This was not possible for AA1 and AA2 due to the lack of suitable clearings, so a weather station was located at Howman's Gap, approximately 600 m from AA1 and 1400 m from AA2, at similar altitude.

### Sap flux

We used the HRM, as developed and presented theoretically by Burgess et al. (2001) and validated by Bleby et al. (2004). For this study, we inserted one probe set at breast height (1.30 m) under the bark of each sample tree, after removing a small section of the bark (about 2–3 cm high and 1-cm wide) to allow probe insertion. SG often has multiple large stems that diverge near ground level (this was commonly the case at SG2 but not SG1); when so, we installed probes only in one stem. An additional five probe sets were also installed at 60^o^ azimuth steps in one tree at three of the four sites (AA1, AA2, and SG1), to assess azimuthal variation in sap flux. Sap flux was averaged for these six sensors (separately for outer and inner probes; see below) unless otherwise noted. Each probe set consisted of three probes, 1.3 mm in diameter and 35 mm in length, spaced 5 mm apart axially in the bole. A drill guide (ICT Intl.) was used to minimize errors in spacing and probe alignment. The center probe contained a heater wire, and the upper and lower probes contained two thermocouples each, located 12.5 and 27.5 mm from the probe hub. The sensors located 12.5 mm from the probe hub comprise the “inner probe” in a probe set, and the sensors at 27.5 mm form the “outer probe.” Heat pulses (40 or 50 J) were triggered by a 16-bit microprocessor unit attached to the tree adjacent to the probes, approximately 10 cm to the side of the probe insertion point, and temperature ratios were recorded 80 sec after each pulse. Measurements were recorded every 30 min. The probe interfaces were connected to ICT SmartLogger data-loggers, powered by one or two 12-volt truck batteries that were continuously recharged by solar panels.

Raw heat pulse velocities were calculated using values of thermal diffusivity (*k* = 2.18–2.32 × 10^–3^ cm^2^ sec^–1^) measured in one tree core at each of the four sites, and corrected for wounding using a homogeneous third-order polynomial (Burgess et al. 2001), assuming a wound diameter of 1.8 mm. The flux of xylem water through sapwood (this is “sap velocity on a total sapwood area basis” in the terminology of Edwards et al. 1997) was calculated by multiplying corrected heat pulse velocities by the quantity *ρ_b_*(*c_w_*+*m_c_c_s_*)/(*ρ_s_c_s_*), where *ρ_b_* is basic density of sapwood, *c_w_* and *c_s_* are heat capacities of dry wood and sap (water), *m_c_* is sapwood moisture content (mass ratio of sap to dry wood) and *ρ_s_* is density of sap (note, this correction equals *ρc*/(ρ_s_*c*_s_), where ρ and *c* are the bulk density and bulk heat capacity of fresh wood, respectively; Marshall 1958; e.g., Burgess et al. 2001). *ρ_b_, c_w_*, and *m_c_* were measured on cores taken from one tree at each site; the resulting conversion factors ranged from 0.701 to 0.759 (dimensionless).

**Figure 1:**
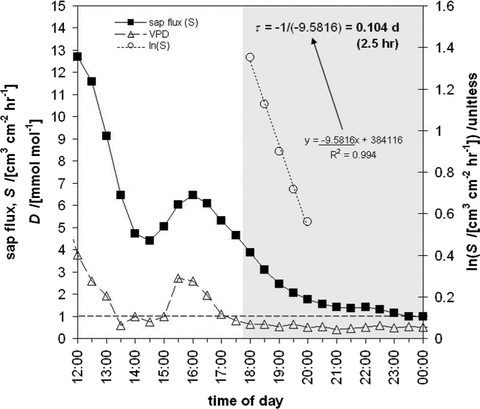
Demonstration of the method used to estimate the refilling time constant, τ. On nights with low evaporative demand (*D* below 1 mmol mol^–1^, indicated by the horizontal dashed line), a line was fitted to the natural logarithm of sap flux (*S*) for the first five points (2.5 h) after sunset. The resulting estimate of τ equals minus the inverse of the regression slope, or 0.104 days (coincidentally also 2.5 h) in the example shown here. The gray area represents the dark period. The data shown are from an outer probe in a *E. delegatensis* tree at site AA1 on the evening of 02 October, 2009.

We could not fell trees or cut through stems to ensure zero-flow for probe calibration because our sites are located on protected national parklands. Instead, we estimated baselines in situ by analyzing periods in which D remained below 0.1 kPa for at least 24 h. The mean heat pulse velocity during the final 25% of the longest such period available for each sensor was taken as the baseline for that sensor. This assumes that refilling of depleted bole water stores is complete within 18 h—an assumption strongly supported by the time-course of sap flux on nights with low *D* (e.g., Fig. 1).

This study was concerned strictly with the nocturnal *fraction* of diel water loss, and not with absolute sap flow or transpiration rates. Therefore, we did not calculate sap flow per se, and we report sap fluxes (cm^3^ cm^–2^ h^–1^) separately for the inner and outer probes in each tree. This allowed us to detect whether the relationship between nocturnal and diurnal water loss differed between the outer and inner sapwood.

Single-point data gaps were filled by linear interpolation between the adjacent points; larger gaps were not filled. For sap flux data, high-frequency noise was reduced by local weighted linear least squares smoothing (loess) with a 5-point window and a tricube weight function.

### Separation of transpiration and refilling

**Figure 2:**
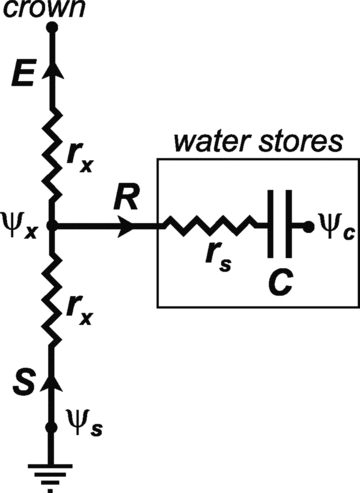
Resistance network that is the basis of the separation method derived in the Appendix and used in this study to separate the transpiration and refilling components (*E* and *R*, respectively) of sap flux (*S*). Soil to crown xylem resistance is 2*r_x_*; bole water stores are modeled as a capacitor connected by a resistance, *r_s_*, to the midpoint of the soil–crown xylem flow path. Water potentials (soil, *ψ_s_*; xylem, *ψ_x_*; water stores, *ψ_c_*) appear in the separation method's derivation but not the final expressions; crown water potential does not appear in the method at all, so it is not labeled here.

We separated the transpiration (*E*) and bole refilling (*R*) components of *S* using a simple flow model based on the resistance network in Figure 2. Details are in the Appendix. Briefly, we assume fluxes across basal sensors and into/out of bole water stores are driven by water potential gradients, which are linked to water content by capacitance (assumed constant). The ratio of storage to xylem resistance (*r_s_* and *r_x_*, respectively) parses the two flows, and appears in the model as a parameter, β = *r_s_*/(*r_s_*+*r_x_*). The model requires one other parameter: the time constant, τ, for refilling in the absence of transpiration (estimation of τ is explained below). β is unknown, so we compared estimates using three values: 0, 0.5, and 1. β = 0.5 represents equal storage and axial resistances, whereas β = 0 implies the water stores are located in the axial flow path itself (so *r_s_* = 0). β = 1 is a degenerate case: *r_s_* is infinitely larger than *r_x_*, so there are no storage flows and *E* = *S*. β is unknown, but two previous studies found β between 0.04 and 0.5 (Phillips et al. 1997; Verbeeck et al. 2007). Computation of *E* is described in the Appendix.

### Estimation of refilling time constants

We estimated τ independently for each sensor (inner and outer) as follows. On each night for which *D* was below 1 mmol mol^–1^ (0.1 kPa) at sunset and for at least 3 h thereafter, we fitted an exponential to sap flux (*S*) versus time (*t*) (*S* = *c*· exp(–*t*/τ), where *c* and τ are constants) by calculating the linear regression of ln(*S*) against *t* (ln(*S*) = (–1/τ) ·*t*+ ln(*c*)) for that time period. The time constant τ was taken as the negative inverse of the regression slope. This procedure is demonstrated in Figure 1. For some probes (one outer and one inner probe at AA2; two outer probes and one inner probe at SG1, all from the intensively probed tree at SG1; and four inner probes at SG2), this procedure failed to yield any estimates of τ; and sap flux data for those probes were excluded from subsequent analysis. The mean of the resulting estimates for each probe was used in all subsequent calculations, and the SE of the estimates was used to gauge τ-related uncertainty in the estimation of *E* from *S*.

### Statistical analyses

All statistical analyses were carried out in SAS Enterprise Guide 4.3.0.10196 (SAS Corp, Cary, NC). To compare the nocturnal fractions of diel water loss (*f_n_*) between probe depths and values of β, we performed mixed nested analyses of variance (ANOVAs). Site was a random effect nested within species, and probe depth (inner vs. outer) and β (0, 0.5, or 1.0) were fixed effects. These analyses were then repeated using site and probe depth as grouping variables rather than effects, and Tukey's LSD post hoc tests were performed to assess differences in mean *f_n_* between the three β values for each site/probe depth combination. We also compared *f_n_* and τ between species and sites based on β = 0.5 alone, using the same ANOVA structure but without the β effect.

We performed multiple linear regressions with nightly mean *E* (*E_n_*) averaged among trees at each site (inner and outer probes separately) as the dependent variable and nightly means of *D, U* and RWC as the independent variables. Regressions were repeated three times, using RWC measured at 5, 20, and 50 cm depths, because a single regression with all three RWCs produced high variance inflation factors, indicating multicollinearity. These regressions invariably showed *D* to be the dominant driver of *E_n_*, with RWC and *U* showing variable and sometimes inconsistent effects, so we sought to clarify the effects of RWC and *U* per se by performing a second set of regressions, this time using *G_n_* (= *E_n_*/*D*) as the dependent variable and only *U* and RWC as independent variables. All variables were log transformed prior to regression to increase normality.

## Results

We collected sap flux and environmental data for an average of slightly over 1 year (377 days) at each site, for 23 trees (six at each site, except five at AA2). This included 503 days for all probes at AA1, 329 days for all probes at AA2, 247–410 days for the probes at SG1, and 142–316 days for the probes at SG2. Because periods of available data differed among sites, statistical comparisons of sites using all available data may be biased. Comparative analyses therefore focused on a single time period of 70 days (12 November, 2009–20 January, 2010) during that data were continuously available for all sites. During that period, mean *T_a_* was 1.7–3.4°C greater at the AA sites than at the SG sites, and ranged from 12.5°C (SG2) to 15.9°C (AA2) (Table 1). RH was greatest at AA1 and least at AA2. Soil moisture profiles differed considerably among sites, with uppermost soil (5-cm depth) having greatest RWC at AA1 and SG1 but the smallest RWC at AA2 and SG2 (Table 1).

### Refilling time constant

Mean τ (±SE) among trees at each site ranged from 2.04 ± 0.32 h (at SG1) to 3.70 ± 0.36 h (SG2) for outer probes, and from 1.55 ± 0.30 h (AA2) to 3.8 ± 0.15 h (SG2) for inner probes (Table 2). Analysis of variance showed that τ was similar between species (2.37 ± 0.19 h for AA vs. 2.69 ± 0.25 h for SG; *P* = 0.65) and probe depths (2.69 ± 0.20 h for outer probes vs. 2.32 ± 0.25% for inner probes; *P* = 0.53), but differed among sites (*P* < 0.0001) (Table 2).

**Table 2:** Refilling time constant, τ (h), estimated from the time-course of sap flux on nights with low evaporative demand, as described in the Methods and demonstrated in Figure 1. Data shown represent means ± SE among trees

Refilling time constant, τ/h			

Site	All probes	Outer probes	Inner probes
AA1	2.8 ± 0.25	2.75 ± 0.24	2.84 ± 0.47
AA2	1.87 ± 0.2	2.18 ± 0.19	1.55 ± 0.3
Both	2.37 ± 0.19	2.49 ± 0.17	2.25 ± 0.34
SG1	2.00 ± 0.21	2.04 ± 0.32	1.95 ± 0.3
SG2	3.73 ± 0.27	3.7 ± 0.36	3.81 ± 0.15
Both	2.69 ± 0.25	2.87 ± 0.34	2.42 ± 0.38

### Separation of transpiration and refilling: analysis of the method

**Figure 3:**
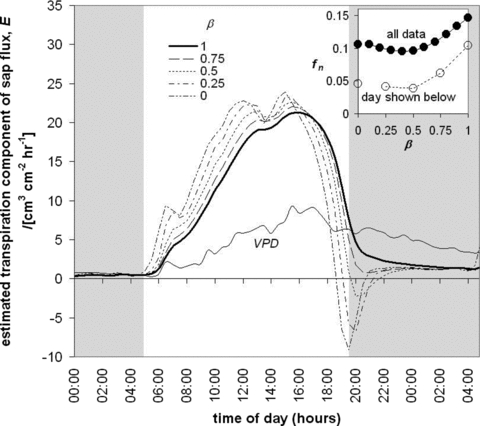
Estimated transpiration component of sap flux for different values of β (as shown in the legend), for one outer probe in a *E. delegatensis* tree at AA1, on 09–10 December, 2009. The thick solid line (β = 1) is sap flux (*S*) itself, because the separation method simply yields *E* = *S* for β = 1. **Inset**: fraction of water lost at night, *f_n_*, in relation to β, for the diurnal and nocturnal period shown here (open symbols) and averaged for all data, over all outer probes at this site (closed symbols). The lines connecting the symbols do not extend to the values shown for β = 0 because *E* for the latter scenario is calculated using a different formula (Equation [A13]).

Figure 3 shows example time courses of *S, E*, and *R* estimated using the method derived in the Appendix for β = 0, 0.25, 0.5, 0.75, and 1, for a single outer probe at AA1 during one 24-h period. Inferred *f_n_* is also shown (inset in Fig. 3) for that probe and time period, and for all outer probes averaged over the entire dataset for AA1. Two patterns are evident. First, inferred *f_n_* was smallest for β≈ 0.4–0.5 and largest for β = 1. Second, for β < 0.75, *E* declined strongly around sunset, to below the value it attained later in the night. For β≤ 0.5 in the example shown in Figure 3, inferred *E* became negative in the peak refilling period (for calculations of *f_n_*, we set *E*≥ 0). A third pattern that is not evident from Figure 3 is that the method becomes numerically unstable at small nonzero β, giving rise to oscillations. For the data shown in Figure 3, these oscillations overwhelmed the signal for 0 < β < 0.08 (β = 0 was calculated using a different algorithm, as described in the Appendix).

### Magnitude of inferred transpiration component of sap flux

**Figure 4:**
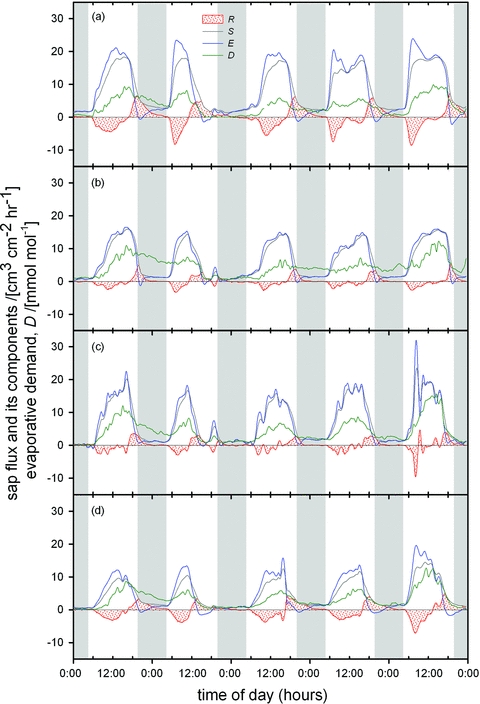
Sample results from each site for a 5-day period (09–13 December, 2009), showing sap flux (gray lines) and its transpiration (blue lines) and refilling components (red lines and red stipling) inferred using the method described in the Appendix, using β = 0.5. Evaporative demand (green lines) is shown for reference. Gray areas indicate dark periods. (A) A 44-cm (DBH) *E. delegatensis* from site AA1, with refilling time constant, τ, of 3.80 ± 0.15 h. (B) A 76-cm *E. delegatensis* from site AA2, τ = 1.63 ± 0.19 h. (C) A 21-cm *E. pauciflora* from site SG1, τ = 1.56 ± 0.24 h. (D) A 27-cm *E. pauciflora* from site SG2, τ = 4.23 ± 0.26 h. These examples are all from outer probes.

**Figure 6:**
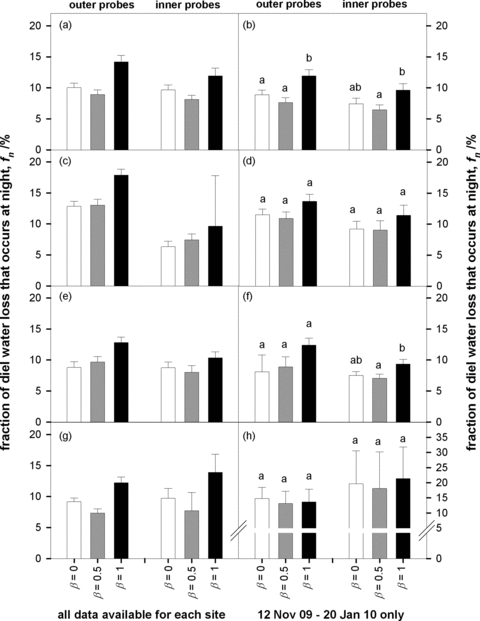
Estimated fraction of diel water loss that occurs at night (*f_n_*/%), calculated using three values of β (0, 0.5, 1.0). For β = 1, *E = S*, so the solid bars represent the nocturnal fraction of diel sap flux. Bars represent means (± SE) of *f_n_* among trees at each site, calculated separately for outer and inner probes. (A and B) AA1; (C and D) AA2; (E and F) SG1; (G and H) SG2. (A, C, E, and G): calculations using all data available for each site; (B, D, F, and H): calculations for data from the 70-day period used for statistical analyses. Within each group of three bars in (B, D, F, and H), different letters indicate significant differences according to Tukey's LSD (*P* < 0.05).

Based on the preceding analysis, we analyzed the rest of our data using three β values (0, 0.5, and 1). Nocturnal sap flux (*S*, which equals *E* for β = 1) ranged from 9.6 to 17.9% of diel sap flux (for inner and outer probes, respectively, both at AA2) (Fig. 6). Figure 4 shows sample estimates of *E* and *R* based on β = 0.5, and the associated *S* measurements, for 5 days at each site. The diel cycles shown in Figure 4 show a common pattern of water being withdrawn from stores in the day (*R* < 0), followed by a period of refilling (*R* > 0) that generally coincides with the decline of *S* in the hours before sunset. In some cases, the period of stored-water withdrawal was sharply punctuated by fluctuations in *S*, apparently driven by *D*. Figure 4 also suggests that refilling sometimes began, and was mostly completed, before sunset, when *D* declined substantially before sunset (e.g., the second day shown for site AA1). Another common pattern in Figure 4 is that inferred *E* drops to zero for a period, usually early in the night and coincident with the greatest rates of refilling (these periods actually represent periods where the separation method yields negative *E*, as described above and shown in Fig. 3).

**Figure 5:**
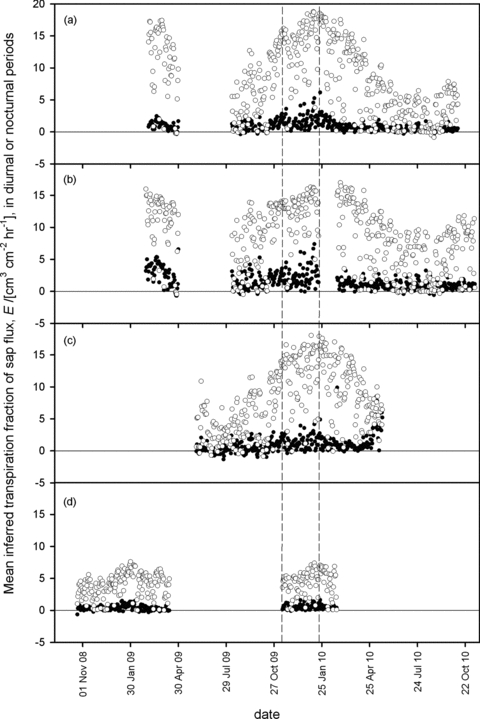
Inferred transpiration component of sap flux, *E*, averaged among trees and probe depths at each site during diurnal and nocturnal periods. Sites: (A) AA1, (B) AA2, (C) SG1, (D) SG2. The dashed lines delimit the 70-day period for that data were continuously available for all sites simultaneously.

Figure 5 shows long-term trends in *E*, averaged among trees at each site, during each diurnal and nocturnal period for which data were available. Mean diurnal *E* ranged from –0.87 (17 October, 2009 at SG1, and 24 August, 2010 at AA1) to 18.8 cm^3^ cm^–2^ h^–1^ (09 January, 2010 at AA1). Mean nocturnal *E* (*E_n_*) ranged from –1.33 (25 April, 2009 at SG1) to 9.89 cm^3^ cm^–2^ h^–1^ (05 March, 2010 at SG1). Note that negative values of inferred *E* arose in this study from two very different causes: overestimation of refilling by the separation method for β < 0.75, discussed above, and negative *S*. Mean nocturnal *S* itself was negative on 5.2% of all nights (over all trees in this study); mean *E_n_* was negative (despite positive mean *S*) on an additional 5.6% of nights. Calibration error could cause spurious negative *S* for individual probes or trees, but is unlikely to explain a consistent pattern of negative mean *S* among trees at each site. It is more likely that negative *S* indicates downward flow from the canopy to the soil, as reported previously (Burgess et al. 1998; Caldwell et al. 1998; Burgess and Dawson 2004; Oliveira et al. 2005; Burgess and Bleby 2006).

### Estimated nocturnal fraction of diel water loss (f_n_)

Estimates of *f_n_* using β = 0, 0.5, and 1 are shown on the right hand side of Figure 6, for the 70-day period used for statistical analysis; the left side of Figure 6 shows estimates of *f_n_* calculated using all available data for each site. Analysis of variance showed *f_n_* was similar between species (*P* = 0.64) and between inner and outer probes (*P* = 0.62), but differed significantly among sites (*P* < 0.0001). Inferred *f_n_* was similar for the two values of β (0 and 0.5) used to separate transpiration from refilling (10.0 ± 0.8% for β = 0, 9.2 ± 0.9% for β = 0.5; *P* = 0.44) but *f_n_* was larger for β = 1, the null scenario in which *E = S* (11.8 ± 0.8%; *P* = 0.02). This indicates that transpiration represented ∼78–85% of nocturnal sap flux during the 70-day period used for statistical analysis. A separate ANOVA on only the β = 0.5 based *f_n_* estimates also showed that *f_n_* was similar between species (8.6 ± 0.6% for AA vs. 9.8 ± 1.7% for SG; *P* = 0.62) and outer and inner probes (*P* = 0.81), but differed among sites (*P* = 0.04).

### Environmental drivers of nocturnal water loss

Multiple regression analysis predictably suggested that *E_n_* (averaged among trees at each site) was strongly correlated with *D* in all sites, for both inner and outer probes (Table 3). For outer probes, correlations between *E_n_* and RWC were significant (*P* < 0.05) only at AA2 and SG2. For inner probes, correlations with RWC at 5- and 20-cm depths for inner probes were significant for AA1, AA2, and SG1, but not SG2; the latter result may reflect the small number of inner probes (two of six) at SG2 for which we were able to estimate τ, and therefore *E_n_*. Correlations with RWC at 50 cm were insignificant at all sites except AA2. Correlations with wind speed (*U*) for outer probes were significant and negative at all sites except SG1, and for inner probes, at both AA sites but neither of SG site.

**Table 3:** Regression slopes from multiple regression analysis of *E_n_* (nocturnal transpiration component of sap flux) in relation to environmental drivers (*D* = evaporative demand; *U* = wind speed; RWC = soil relative water content), for all variables averaged for each nocturnal period and among trees in each site. Regressions were repeated three times, using RWC values measured at three different depths, as indicated in the left column. Slopes were standardized by subtracting the mean and dividing by the standard deviation for each variable. Significance levels are as follows: ***P < 0.001; **P < 0.01; *P < 0.05; ns, *P* > 0.05). Sample sizes were as follows. Outer probes: AA1, *n* = 475; AA2, 323; SG1, 198; SG2, 278. Inner probes: AA1, 562; AA2, 325; SG1, 205; SG2, 174

		Standardized slopes for regressions on *E_n_*					
		
RWC depth		Outer probes			Inner probes		
			
	Site	*D*	*U*	RWC	*D*	*U*	RWC
5 cm	AA1	0.913***	–0.107***	ns	0.763***	–0.073*	–0.094*
	AA2	0.932***	–0.133*	0.132*	0.951***	–0.113*	0.202**
	SG1	1.045***	ns	ns	1.064***	ns	0.182*
	SG2	0.77***	–0.235***	0.282***	0.552***	ns	ns
20 cm	AA1	0.865***	–0.143***	ns	0.729***	–0.13***	–0.11**
	AA2	0.915***	–0.134*	0.148**	0.921***	–0.113*	0.205***
	SG1	1.047***	ns	ns	1.068***	ns	0.184*
	SG2	0.743***	–0.232***	0.326***	0.542***	ns	ns
50 cm	AA1	0.879***	–0.143***	ns	0.778***	–0.134***	ns
	AA2	0.914***	–0.138*	0.174**	0.917***	–0.116*	0.226***
	SG1	0.982***	ns	ns	1.023***	ns	ns
	SG2	0.724***	–0.223***	0.329***	0.537***	ns	ns

The effects of *U* and RWC were much smaller than those for *D* (standardized regression slopes averaged 0.86 for *D*; cf. –0.11 and +0.13 for *U* and RWC, respectively; Table 3). Additionally, these effects may have been biased by correlation between RWC and *D*: variance inflation factors for *D* and RWC at 5 cm were around 2.0, indicating moderate multicollinearity. We attempted to identify the individual roles of *U* and RWC separate from that of *D* by regressing *G_n_* (= *E_n_/D*) against RWC and *U* (Table 4). That analysis found strong correlations between *G_n_* and RWC at 5 cm in all sites except SG2, but with RWC at 20 and 50 cm depths only at AA1 and SG1. Wind speed was negatively correlated with *G_n_* at AA1 and positively correlated with *G_n_* at SG2 in most analyses; the latter effects were more evident for inner probes. No other sites showed significant correlations between *G_n_* and *U*.

**Table 4:** Regressions slopes from multiple regression analysis of *G_n_* (nocturnal transpiration component of sap flux, *E_n_*, divided by evaporative demand, *D*) in relation to environmental drivers (*U* = wind speed; RWC = soil relative water content), with each variable averaged for each nocturnal period and among trees in each site. Regressions were repeated three times, using RWC values measured at three different depths, as indicated in the left column. Slopes were standardized by subtracting the mean and dividing by the standard deviation for each variable. Significance levels are as follows: ***P < 0.001; **P < 0.01; *P < 0.05; ns, *P* > 0.05). Sample sizes were as follows. Outer probes: AA1, *n* = 337; AA2, 314; SG1, 209; SG2, 40. Inner probes: AA1, 256; AA2, 313; SG1, 211; SG2, 220

		Standardized slopes for regressions on *G_n_* (*E_n_/D*)			
		
RWC depth		Outer probes		Inner probes	
			
	Site	*U*	RWC	*U*	RWC
5 cm	AA1	–0.239***	0.425***	–0.205***	0.476***
	AA2	ns	0.329*	ns	0.294*
	SG1	ns	0.69***	ns	0.409*
	SG2	ns	ns	0.25*	ns
20 cm	AA1	–0.309***	0.25***	–0.233***	0.407***
	AA2	ns	ns	ns	ns
	SG1	ns	0.788***	ns	0.475*
	SG2	ns	ns	0.287**	ns
50 cm	AA1	–0.298***	0.216***	–0.207***	0.355***
	AA2	ns	ns	ns	ns
	SG1	ns	0.768***	ns	0.441*
	SG2	0.346*	ns	0.294**	ns

### Azimuthal variation in the nocturnal fraction of water loss

**Figure 7:**
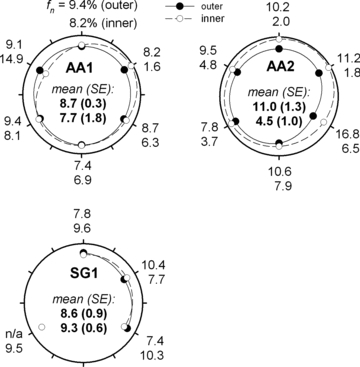
Azimuthal variation in inferred nocturnal fraction of diel water loss (*f_n_*/%) for one tree at each of three sites (AA1, AA2, and SG1) that was instrumented with six probe sets installed at 60^o^ azimuth steps. Values shown are oriented as a compass (top = north). *f_n_* is graphed as radial function (zero at the outer margin, 50% at the center), for outer probes (solid circles and lines) and inner probes (open circles and broken lines). Missing data for 180^o^, 240^o^, and 300^o^ at SG1 represent sensors for which the refilling time constant, τ, could not be estimated due to the lack of suitable data.

Variation in *f_n_* among the six probes installed at 60^o^ azimuth steps in one tree at AA1, AA2, and SG1 was greater for inner than outer probes at AA1 and AA2 (coefficients of variation [cv = SD/mean] were 0.09 and 0.28 for outer probes vs. 0.56 and 0.55 for inner probes), but not at SG1 (cv = 0.19 vs. 0.12 for outer vs. inner probes). *f_n_* for inner probes at AA1 and AA2 showed a more pronounced eccentricity, being smallest for the northeastern probes in both cases (Fig. 7); data were unavailable for two azimuths at SG1, so eccentricity could not be clearly assessed.

## Discussion

### Nocturnal water loss estimated from sap flux

Both subalpine eucalypt species in our study showed substantial nocturnal water loss: *f_n_* averaged 8.6% for AA and 9.8% for SG. These values are in the range (0–20%) reported for 27 woody species from a wide range of habitats by Dawson et al. (2007), and are similar to values reported for *Betula papyrifera* by Daley and Phillips (2006) (8.6–13.5%). However, in comparison with available data for Australian native tree species from warmer regions that experience less extreme seasonal climate variation than our subalpine sites, these estimates of *f_n_* are slightly higher (cf. 5–7% for *E. argophloia, E. camaldulensis, E. dunnii, E. globulus, E. grandis, E. occidentalis, E. sideroxylon*, and *E. tereticornis*, Phillips et al. 2010; 6–8% for *E. parramattensis* and *A. bakeri*, Zeppel et al. 2010). The means for *f_n_* reported above were estimated during a 70-day period in spring and summer; however, means for the entire dataset, which included winter and autumn periods for each site, were in the same range (9.6% for AA and 8.6% for SG).

AA and SG did not differ significantly in *f_n_* despite the two species’ very different size and ecology. SG forms patchy stands in flatter terrain at higher elevations than AA and resprouts basally, whereas AA is approximately three times taller at our sites (40–50 m, vs. 12–20 m), forms continuous stands on steep terrain at slightly lower elevations and sprouts only from seed. Our results do not support our hypothesis that the much larger species, AA, would exhibit more conservative nocturnal water loss than smaller SG. That hypothesis was based on generic findings of declining stomatal conductance with increasing tree height (Saliendra et al. 1995; Bond and Kavanagh 1999; Hubbard et al. 1999; Delzon et al. 2004; Ryan et al. 2006). Phillips et al. (2010) also found similar *f_n_* among eight *Eucalyptus* species ranging from 6 to 14 m in height at the time of measurement, which, together with our data, suggests height does not constrain or determine *f_n_* in eucalypts. More direct tests are needed and might, for example, compare *f_n_* among individuals of the same species along a chronosequence.

### Variation in f_n_ was dominated by between-site differences

**Figure 8:**
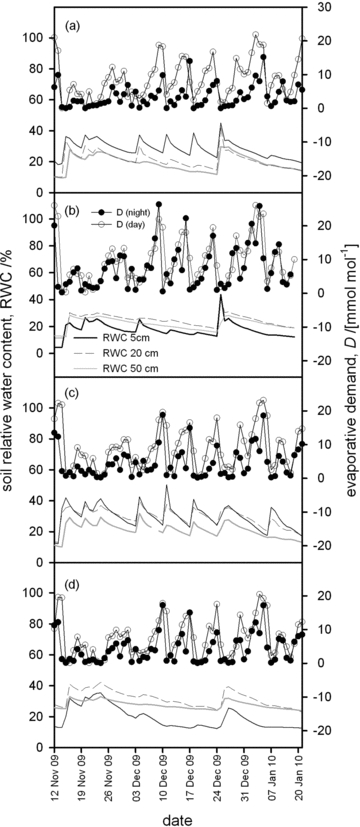
Soil relative water content, RWC, measured at three depths (5, 20, and 50 cm) and evaporative demand, *D* (vapor pressure mole fraction deficit of the air), measured in each site and averaged during diurnal and nocturnal periods, for the 70-day period for that data were continuously available for all sites simultaneously. Sites: (A) AA1, (B) AA2, (C) SG1, (D) SG2.

We measured sap flux in two contrasting sites for each species, and found remarkable differences between sites. For example, mean *f_n_* among trees was 39% greater at AA2 than AA1, and 92% greater at SG2 than SG1. The cause of this variation is uncertain. AA2 is on a moderate north-facing (sun-facing) toe slope, while the AA1 site is nearly flat (Table 1). Yet, SG1 has a more pronounced slope than SG2. The differences in *f_n_* could have been partly due to microclimate differences among these sites, despite their proximity. For example, AA1 and SG1 both received a series of small rainfall events during the middle of the 70-day window used for statistical comparisons among sites (as reflected in the 5-cm soil RWC data in Fig. 8), but these events were much smaller or absent at AA2 and SG2. AA2 and SG2 also differed from AA1 and SG1 in having lower RWC near the soil surface (5 cm) than at 20 and 50 cm, and in showing no correlations between *G_n_* (*E_n_/D*) and soil RWC at 20 or 50 cm; the reverse was true for AA1 and SG1. Zeppel et al. (2010) also found correlations between *E_n_* and RWC in shallow soil layers (<60 cm) but not for deeper soil. These results suggest the effect of soil moisture on *E_n_* may be sensitive to site conditions, including factors that affect the vertical moisture profile. At a minimum, our results suggest generalizations of *f_n_* from measurements at a single site should only be made with a high degree of caution.

### f_n_ varied with azimuth of probe insertion

We found substantial variation in *f_n_* with aspect (azimuth) of probe insertion, with cv (SD/mean) ranging from 9–28% for outer probes and 12–56% for inner probes among six probes at 60^o^ azimuth steps. Interestingly, azimuthal variation was greater for inner than outer probes, despite the lack of a statistically significant difference in *f_n_* between inner and outer probes in the whole dataset. Other authors have also reported substantial azimuthal variation in sap flux (e.g., Hatton et al. 1995; Zang et al. 1996; Vertessy et al. 1997). To our knowledge this is the first report of azimuthal variation in the nocturnal fraction of sap flux or water loss. This highlights the need to characterize positional variation in sap flux, as argued by other authors (Hatton et al. 1995). Eucalypts in particular may have striking asymmetry in their crowns, and an obvious, yet still untested, speculation is that the azimuthal distribution of leaf area could be reflected in azimuthal sap flux measurements.

### Evaporative demand was the dominant environmental correlate of nocturnal water loss

*D* was clearly the strongest environmental correlate of *E_n_* across all sites, consistent with most other reports (Dawson et al. 2007; Fisher et al. 2007; Phillips et al. 2010; Zeppel et al. 2010). Soil moisture (RWC) was a significant driver in some sites but not others. However, wind speed (*U*) was a significant negative correlate of *E_n_* at most sites. These results were somewhat obfuscated by correlation between *D* and RWC. A separate assessment of the roles of *U* and RWC in regulation of *E_n_* by stomatal and boundary layer effects (using *G_n_* = *E_n_/D* as a proxy for crown conductance), suggested a positive effect of *U* on *G_n_* at SG2 and a negative effect at AA1. The lack of a clear, strong effect of *U* contrasts with the results of Phillips et al. (2010), but is consistent with results of Zeppel et al. (2010) and Fisher et al. (2007). It is unclear how increased *U* could reduce *E_n_* or *G_n_*, although there are reports of stomatal closure in direct response to high wind speeds (e.g., Grace et al. 1975; Gutierrez et al. 1994; Campbell-Clause 1998), albeit not for large trees in native forests, as far as we are aware. Soil RWC at 5 cm was a significant positive correlate of *G_n_* in most sites, consistent with the general observation that nocturnal stomatal opening is suppressed by low soil water supply (Dawson et al. 2007; Zeppel et al. 2010).

### A dynamic method to separate transpiration and refilling

We developed a novel method to separate the transpiration and refilling components of sap flux. Some previous studies used a finite time interval after sunset during which sap flux was presumed to represent refilling (Fisher et al. 2007; Phillips et al. 2010), which focuses on the duration of refilling—an extensive property of the bole's water status that may differ from one night to another. Our method is a step forward in that we quantify the time constant for refilling, which is an intensive property of the bole's hydraulic structure and may therefore be more conservative. For example, the duration of refilling may be quite large on nights that follow days of high water loss, but negligible following days when water loss is suppressed by low *D* late in the day (e.g., cf. the first two nights shown in Fig. 4). Applying a fixed refilling period would not distinguish these scenarios, and could bias relationships among inferred nocturnal and diurnal water loss and environmental factors. Additionally, our method infers not only integrals, but also time-courses of water loss, which opens the door to further studies of mechanistic controls on crown water use.

Our method requires values for two parameters: τ, the refilling time constant, and β, the ratio of storage resistance to storage plus xylem resistance. We estimated τ by fitting exponentials to sap flux versus time on nights with low *D* (Fig. 1). A similar approach was employed by Chuang et al. (2006). More generally, approaches to estimating hydraulic time constants in branches have been discussed elsewhere (e.g., Phillips et al. 1997, 2004) and can be adapted to small trees. Our estimates for τ (2.4 h for AA, 2.7 h for SG) are somewhat larger than most estimates for other species: cf. 0.7 h for *Pinus taeda* of mean height 7.1 m and mean diameter at breast height (DBH) 9.4 cm (Phillips et al. 1997); 1.2 h for a 6.7-m tall *Picea abies* tree (Chuang et al. 2006), 0.50–0.56 h for four tropical forest trees ranging in height from 22 to 38 m and DBH from 34 to 98 cm (Meinzer et al. 2004), and 2.3 h for a small desert shrub, *Encelia farinosa* (Hunt and Nobel 1987). As *D* rarely reached zero at our sites, we set a low *D* threshold of 1.0 mmol mol^–1^ (0.1 kPa) to identify nights on which to estimate τ from the relaxation kinetics of sap flux. Nonzero transpiration rates on those nights could have caused overestimation of τ due to conflation of refilling with declining transpiration.

Few data are yet available with which to estimate β. A modeling analysis on *P. taeda* (Phillips et al. 1997) gave a mean β of 0.042 and suggested that β increased linearly with DBH (note our β is the inverse of β as estimated by Phillips et al. 1997). A similar analysis by Verbeeck et al. (2007) on *P. sylvestris*, in which *r_s_* was estimated from the literature and *r_x_* was calibrated, led to mean β of 0.49 (our calculations, based on data in their paper). Our analysis across a range of values for β indicated that inferred *f_n_* was smallest for intermediate β (0.4–0.5). At β = 0 the storage resistance is zero, which may accelerate modeled refilling; indeed, for our data, low β values led to physically unrealistic inferences of negative transpiration rates even when *D* was substantial. Thus, at low β the method overestimated refilling and underestimated transpiration for some sites. This could indicate systematic overestimation of τ, as discussed above. It could also arise from omission of the rate of change of soil water potential (*ψ_s_*) from the method's derivation (Equation A11). Equation (A10) suggests that *E* would increase if declining *ψ_s_* were accounted for, provided β < 1, and that this effect would be greater for smaller β. Thus, excluding a real decline in *ψ_s_* should cause underestimation of *E* during refilling. Overestimation of refilling could also arise from decreasing hydraulic resistances, which our method assumes constant. This is plausible if xylem resistance declines during refilling due to repair of embolisms formed during the preceding day.

To ensure our estimates of *f_n_* were conservative, we reported values based on β = 0.5. However, when all available data were pooled among sites, calculated *f_n_* was similar whether we used β = 0.5 or β = 0 (9.2 ± 0.9% vs. 10.0 ± 0.8%, respectively). Inspection of *f_n_* estimates made for site AA1 using a wider range of β values (inset, Fig. 3) suggests furthermore that *f_n_* is relatively robust to variation in β below β≈ 0.75. We therefore suggest that our method may be used to generate high and low reasonable estimates for *f_n_*, using β = 0 and 0.5, respectively. Our approach circumvents the technical difficulty and cost of installing probes in branches or below the main crown (which is not feasible for many trees), as well as the challenges of implementing and parameterizing much more rigorous mathematical approaches (e.g., Tyree 1988; Chuang et al. 2006; Verbeeck et al. 2007). We note that our method is computationally compact and can be applied to measurements of sap flux made by any of the many currently available technologies. On balance, we believe our method—which is based on a novel analytical simplification of tree water flow based on the parameter β, the ratio of storage resistance to storage plus xylem resistance—provides a new and simple approach for probing dynamics of water fluxes in canopies, and is an advance worthy of further study and application.

## Conclusions

We found evidence of substantial nocturnal water loss (*E_n_*) in two eucalypt species, *E. delegatensis* and *E. pauciflora*, that dominate upper elevations in montane regions of southeastern Australia. Our analysis suggested that approximately 9% of water loss occurs at night in these species (cf. approximately 12% of sap flux), and that this proportion differs more among sites for either species than between species or sapwood depths. Evaporative demand was the strongest predictor of *E_n_*, followed by soil moisture. Surprisingly, wind speed was negatively correlated with *E_n_* at most sites and with *G_n_* (*E_n_/D*) at one site, but positively correlated with *G_n_* at only one site.

Our estimates of *E_n_* were based on a novel method for separating the transpiration and refilling components of sap flux, in which water flows are dynamically simulated using a simplified model. The method requires only two parameters and can easily be implemented on data using compact algorithms. A further simplification of this method requiring only one parameter (the time constant for refilling bole water stores) was found to produce similar results, and we believe it represents a promising advance.

## Appendix

### Separation of sap flux into transpiration and refilling components

Here we derive a mathematical technique to separate basal sap flux (*S*) into components due to crown transpiration (*E*) and water flow into bole water stores (*R*, refilling), given measurements of *S* and estimates of two parameters: the time constant (τ) for recharge of bole water stores in the absence of transpiration (*E* = 0), and the ratio (β) of storage resistance (*r_s_*) to the sum of *r_s_* and xylem resistance (*r_x_*): β = *r_s_*/(*r_s_*+*r_x_*). This derivation is based on the resistance/capacitance diagram in Fig. 2. By conservation of mass, *R* equals *S* minus *E*:

(A1)

Sap flux equals the difference between water potentials in the soil (*ψ_s_*) and in the conducting stem xylem (*ψ_x_*), divided by the xylem resistance (*r_x_*):

(A2)

The rate of change of sap-flow if *r_x_* is constant is thus

(A3)

where the overdot signifies differentiation with respect to time. The rate of water flow into bole water stores equals the difference between *ψ_x_* and the water potential of the storage compartment (*ψ_c_*, where the *c* stands for *capacitor*), divided by the storage resistance (*r_s_*):

(A4)

We further assume that *ψ_c_* is negatively proportional to the relative water loss of the storage compartment (RWL = [*W_sat_− W*]/*W_sat_*, where *W* and *W_sat_* are the actual and saturated water content per unit sapwood area, respectively; cm^3^ H_2_O cm^–2^ sapwood); in other words, we assume capacitance of the storage compartment (*C* = –*dRWL*/*dψ_c_*; MPa^–1^) is constant. This leads to

(A5)

Combining Eqns (A1), (A2) and (A4) gives an expression for *E*:

(A6)

Applying A5 to A6 and rearranging yields:

(A7)

We define τ = (*r_s_*+*r_x_*)*CW_sat_*; note that *r_s_CW_sat_* = β. (We show below that τ is the time constant for refilling.) This gives

(A8)

The rate of change of *E* is then

(A9)

The first two terms at right can be written in terms of the rate of change of *S* (Eqn A3), and the third term can be rewritten in terms of *S* and *E* (Eqn (A1)), recognizing that the rate of change of *W* equals the refilling rate *R* (= *S*–*E*):

(A10)

Provided the dynamics of *ψ_s_* are small compared to those of *ψ_x_* and *ψ_c_* (as they mostly are), then the second term in A10 may be ignored, leading to a differential equation for *E*:

(A11)

Eqn (A11) can be converted to a finite difference equation and evaluated in each measurement time interval to reconstruct the time-course of *E* from that of *S*, given estimates of τ and β(see *Numerical procedures* below).

### Limiting cases of β = 0 and 1

Eqn (A11) requires an estimate of the parameter β, which may not be available. Therefore, one would like to assess the impact of wide variation in β on the predictions of Eqn (A11). However, Eqn (A11) is undefined for β = 0, and its application to any real, temporally discrete data must become unstable as β approaches zero. In the limit of β = 0, Eqn (A11) reduces to an algebraic equation for *E* with one parameter, τ:

(A12)

(to see this, multiply both sides of A11 by β and apply β = 0). The derivative of *S* can be estimated by central difference about the current time-step: , where the subscripts *i, i*− 1, and *i*+ 1 denote the current, preceding and following time-steps, respectively, and *δt* is the time interval between time-steps. This gives

(A13)

At the other extreme, β = 1, Eqn (A11) reduces to

(A14)

which can only be true if *E* = *S* and therefore *R* = 0. Thus, β = 1 is equivalent to assuming all nocturnal sap flux is transpiration rather than refilling.

### Numerical procedures

We estimated *E* from *S* using three different values of β (0, 0.5 and 1.0). For β = 0, we reconstructed *E* using Eqn (A13), which is based on central differencing. For β = 1, we simply set *E* = *S*. For 0 > β > 1, it was necessary to solve an ordinary differential equation for *E* (Eqn A11). Second- and higher-order methods commonly used to integrate differential equations are difficult to apply when the equations are driven by discrete data (sap flux, *S*, in this case), without interpolating (downscaling) those data. However, approximating the derivatives in Eqn (A11) by first-order *forward* differences (the Euler method) is asymmetrical, and it is unstable for small β. We therefore computed two estimates for each finite change in *E*, based respectively on forward and backward differences (Eqns (A15) and (A18), derived below), and averaged the two estimates to implement Eqn (A11). We initialized solutions by setting *E* = *S* in the first time step of each data series; results did not differ from an alternative initialization, *E* = 0, after a few time steps.

For the forward interval,  and . Applying these to A11 gives the “forward” estimate of *E_i+1_*:

(A15)

For the backward interval (that preceding the current time-step),  and , which, when applied to Eqn (A11), leads to

(A16)

Eqn (A16) is an implicit expression for *E_i_*, because *E_i_* also appears on the right; thus, this approach is often called “implicit differencing.” Solving for *E_i_* gives

(A17)

Since *i* is arbitrary, it may be replaced with *i*+ 1 to give the “backward” estimate of *E_i+1_*:

(A18)

### Demonstration that τ is the refilling time constant

In the absence of transpiration, *S* = *R*, and both rates equal the difference between soil and storage water potentials divided by the sum of xylem and storage resistances:

(A19)

Eqn (A19) also explicitly recognizes that *R* equals the rate of change of *W*. Applying Eqn (A5) to eliminate *ψ_c_* gives

(A20)

This is a first-order differential equation with respect to *W*. If *ψ_s_, r_x_, r_s_* and *C* are constant, it is easily shown that Eqn (A20) leads to a solution of the form

(A21)

where τ = (*r_x_*+*r_s_*)*CW_sat_*. Thus, τ as defined below Eqn (A7) is the time constant for relaxation of *R* (and *S*) in the absence of transpiration.
